# A kryptoracemic salt: 2-{[2,8-bis­(tri­fluoro­meth­yl)quinolin-4-yl](hy­droxy)meth­yl}piperidin-1-ium (+)-3,3,3-tri­fluoro-2-meth­oxy-2-phenyl­propanoate

**DOI:** 10.1107/S2056989016008495

**Published:** 2016-05-27

**Authors:** James L. Wardell, Solange M. S. V. Wardell, Edward R. T. Tiekink

**Affiliations:** a Fundaçaö Oswaldo Cruz, Instituto de Tecnologia em Fármacos-Far Manguinhos, 21041-250 Rio de Janeiro, RJ, Brazil; b CHEMSOL, 1 Harcourt Road, Aberdeen AB15 5NY, Scotland; c Research Centre for Crystalline Materials, Faculty of Science and Technology, Sunway University, 47500 Bandar Sunway, Selangor Darul Ehsan, Malaysia

**Keywords:** crystal structure, salt, krypto­racemate, hydrogen bonding

## Abstract

The asymmetric unit of the title salt, C_17_H_17_F_6_N_2_O^+^·C_10_H_8_F_3_O_3_^−^, comprises two piperidin-1-ium cations and two carboxyl­ate anions. The cations, each having an l-shaped conformation owing to the near orthogonal relationship between the quinolinyl and piperidin-1-ium residues, are pseudo-enanti­omeric. The anions have the same absolute configuration but differ in the relative orientations of the carboxyl­ate, meth­oxy and benzene groups. Arguably, the most prominent difference between the anions occurs about the C_q_—O_m_ bond as seen in the C_c_—C_q_—O_m_—C_m_ torsion angles of −176.1 (3) and −67.1 (4)°, respectively (q = quaternary, m = meth­oxy and c = carboxyl­ate). The presence of O_h_—H⋯O_c_ and N_p_—H⋯O_c_ hydrogen bonds leads to the formation of a supra­molecular chain along the *a* axis (h = hy­droxy and p = piperidin-1-ium); weak intra­molecular N_p_—H⋯O_h_ hydrogen bonds are also noted. Chains are connected into a three-dimensional architecture by C—H⋯F inter­actions. Based on a literature survey, related mol­ecules/cations adopt a uniform conformation in the solid state based on the letter *L*.

## Abstract 2

The l-shaped cations in the title compound are related across a non-crystallographic centre of inversion and therefore, are kryptoracemic. Supra­molecular chains arise in the crystal packing as a result of O—H⋯O and N—H⋯N hydrogen bonding.

## Chemical context   

Biological considerations remain as the primary reason for the study of mefloquine, Scheme 1, and derivatives thereof. For example, when the racemic compound is protonated (employing HCl as the acid) at the piperdinyl-N atom, the resulting [(R*,S*)-(2-{[2,8-bis­(tri­fluoro­meth­yl)quinolin-4-yl](hy­droxy­meth­yl)piperidin-1-ium chloride salt, usually referred to as racemic *erythro*-mefloquine hydro­chloride, is an anti-malarial drug (Maguire *et al.*, 2006 ▸). Other biological activities have been described for these compounds, namely anti-bacterial (Mao *et al.*, 2007 ▸), anti-mycobacterial (Gonçalves *et al.*, 2012 ▸) and anti-cancer (Rodrigues *et al.*, 2014 ▸).
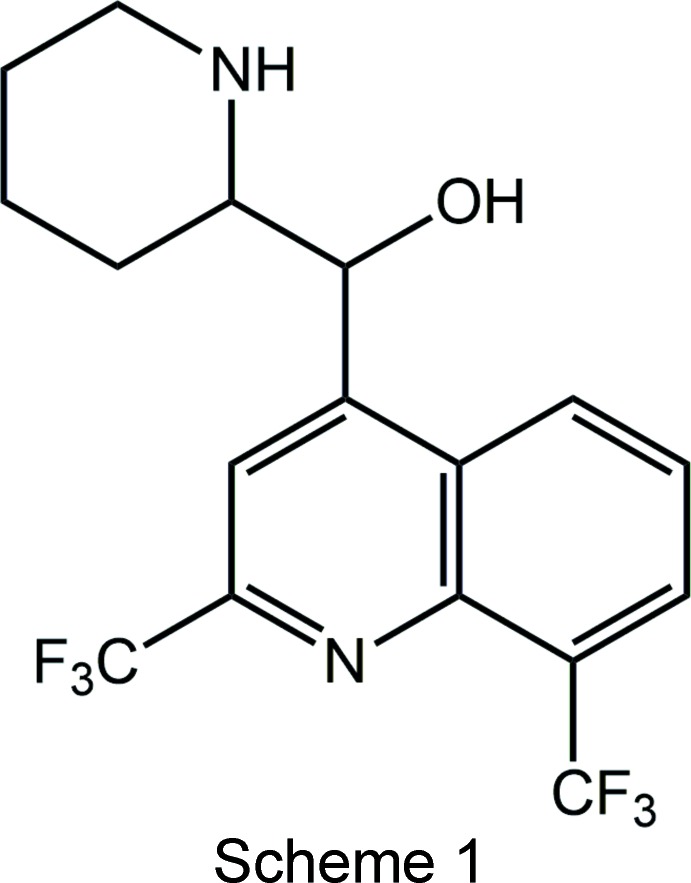
 
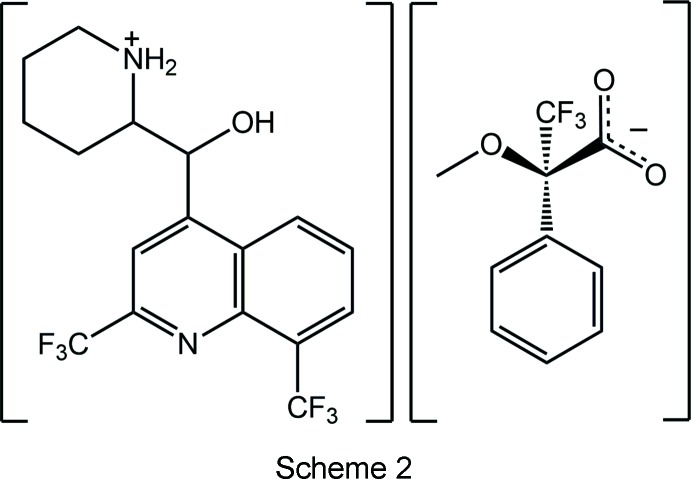


It was in this context that the title salt was isolated from the attempted chiral resolution of mefloquine with the carb­oxy­lic acid, (+)-PhC(CF_3_)(OMe)CO_2_H. Resolution of racemic bases into the individual enanti­omers has been traditionally achieved *via* salt formation with a chiral acid, since usually such salts of the different enanti­omeric bases will have different properties, especially solubilities arising from differences in their crystal structures. Hence, fractional crystallization of such salts is frequently a convenient way to separate the enanti­omers. Crystallography showed the triclinic *P*1 crystals to comprise the [(+)-*erythro*-mefloquinium] and [(−)-*erythro*-mefloquinium] cations with two independent (+)-3,3,3-tri­fluoro-2-meth­oxy-2-phenyl­propanoate anions providing the charge balance, Scheme 2. There is a non-crystallographic enanti­omeric relationship between the cations so the sample is classified as a kryptoracemate. Surveys of this phenomenon have appeared in recent times for both organic (Fábián & Brock, 2010 ▸) and metal-organic (Bernal & Watkins, 2015 ▸) systems. Herein, the crystal and mol­ecular structures of the title salt, (I), are described.

## Structural commentary   

In the present study, the reaction of racemic (±)-erythro-mefloquine with the chiral carb­oxy­lic acid, (+)-PhC(CF_3_)(OMe)CO_2_H, was carried out. However, as revealed by the X-ray crystal structure determination described herein, the isolated crystalline salt contained both mefloquinium enanti­omers and two independent carboxyl­ate anions. It is noticeable that in the ^1^H NMR spectrum in DMSO solution of the isolated crystals, the proton signals, H5, H6 and H7, of the quinolinyl ring are doubled, *e.g*. at δ 7.80 and 7.85 (H6), 8.36 and 8.37 (H5) and 8.85 and 8.89 (H7) p.p.m., suggesting that the quinolinyl fragments in the complex salt are experiencing two slightly different magnetic environments. This doubling is not found for racemic mefloquinium salts of non-chiral acids, such as acetic and nitro­benzoic acids.

**Figure 1:**
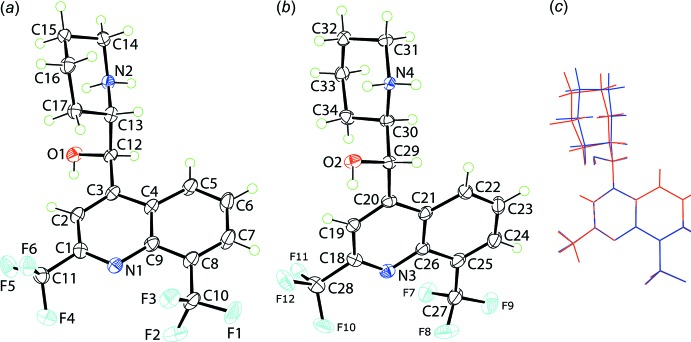
The mol­ecular structures of the (*a*) first and (*b*) second independent cations in (I), showing the atom-labelling scheme and displacement ellipsoids at the 50% probability level. (*c*) An overlap diagram highlighting the similarity of the conformations of the first (red) and inverted second (blue) independent cations. The cations have been overlapped so the the quinolinyl rings are coincident.

**Figure 2:**
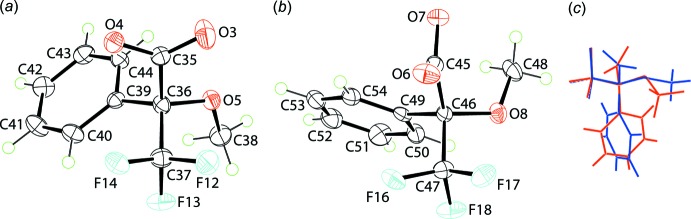
The mol­ecular structures of the (*a*) first and (*b*) second independent anions in (I), showing the atom-labelling scheme and displacement ellipsoids at the 50% probability level. (*c*) An overlap diagram highlighting the differences in the conformations of the first (red) and second (blue) independent anions. The anions are overlapped so the α-atoms about the chiral centre are coincident.

The crystallographic asymmetric unit of (I) comprises two independent mefloquinium cations, Fig. 1 ▸, and two independent carboxyl­ate anions, Fig. 2 ▸. Confirmation of protonation and the formation of a piperidin-1-ium cation is found in the pattern of hydrogen-bonding inter­actions, as discussed in *Supra­molecular features* below. On the other hand, confirmation of deprotonation of the carb­oxy­lic acid during crystallization is seen in the virtual equivalence of the C35—O3,O4 [1.231 (5) and 1.255 (5) Å] and C45—O6,O7 [1.239 (5) and 1.257 (6) Å] pairs of bond lengths.

The N1-containing cation, Fig. 1 ▸ *a*, with *R*- and *S*-configurations at the C12 and C13 chiral centres, respectively, is assigned as [(+)-erythro-mefloquinium], while with inverted configurations at the C29 and C30 centres, respectively, Fig. 1 ▸ *b*, the N3-containing cation is [(−)-erythro-mefloquinium]. The cations are related by a pseudo centre of inversion and indeed the N1-containing mol­ecule is virtually superimposable upon the mirror image of the N3-mol­ecule, Fig. 1 ▸ *c*, with the r.m.s. difference for bond distances and angles being 0.0082 Å and 0.550°, respectively (Spek, 2009 ▸). Differences relate to the relative orientation of the piperidin-1-ium residue. The hydroxyl-O and ammonium-N atoms lie to the same side of the cation being gauche across the methine-C—C(methine) bond with N⋯O = 2.677 (3) Å and O1—C12—C13—N2 = −57.5 (4)° for the N1-cation, with the equivalent values for the N3-cation being 2.734 (3) Å and 59.2 (3)°. Despite the close separation, the O and N atoms are connected by only a weak intra­molecular hydrogen bond as the relevant H atom forms a strong inter­molecular hydrogen bond in each case (see below). The piperidin-1-ium residue lies almost orthogonal to the quinolinyl residue with the C2—C3—C12—C13 and C19—C20—C29—C30 torsion angles being 100.5 (4) and −105.1 (4)°, respectively. Overall, the shape of each cation is based on the letter, *L*.

The non-crystallographic enanti­omeric relationship between the cations is an example of kryptoracemic behaviour, a phenomenon known in both organic (Fábián & Brock, 2010 ▸) and metal-organic (Bernal & Watkins, 2015 ▸) crystals. While known, this is rare occurring in 0.1% of all possible organic structures. This is consistent with the fact that racemic compounds, achiral mol­ecules and those with meso symmetry prefer to crystallize about a centre of inversion.

The anions in (I) have the same absolute structure but differ in terms of the relative orientations of most of the substituents, Fig. 2 ▸ *a*, *b*. As illustrated in the overlap diagram, Fig. 2 ▸ *c*, while the C*C_3_O tetra­hedron is, as expected, virtually superimposable, except for the trifluromethyl groups, the remaining substituents are orientated differently. The differences are qu­anti­fied in the following terms. While to a first approximation the carboxyl­ate and meth­oxy groups lie on a plane in the first anion, Fig. 2 ▸ *a*, [the O3,O4—C35—C36—O5 torsion angles are −18.6 (5) and 162.9 (3)°, respectively, and C35—C36—O5—C38 is −176.1 (3)°], in the second anion, Fig. 2 ▸ *b*, these groups do not lie in a plane [the O6,O7—C45—C46—O8 torsion angles are −112.9 (4) and 65.1 (4)°, respectively, and C45—C46—O8—C48 is −67.1 (4)°]. In addition, the benzene rings occupy different relative positions to the carboxyl­ate groups as indicated in the C_6_/CO_2_ dihedral angles of 89.1 (2) and 77.91 (17)° respectively.

## Supra­molecular features   

**Figure 3:**
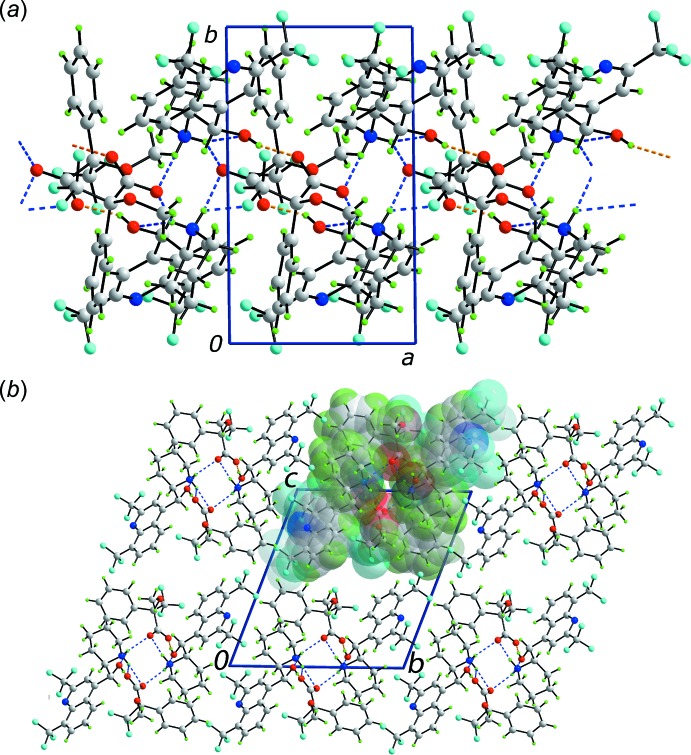
The mol­ecular packing in (I), showing (*a*) a view of a supra­molecular chain aligned along the *a* axis and (*b*) a view in projection down the *a* axis of the unit-cell contents showing the stacking of supra­molecular chains; one chain has been highlighted in space-filling mode. The O—H⋯O and N—H⋯O hydrogen bonds are shown as orange and blue dashed lines, respectively. Colour code: F, cyan; O, red; N, blue; C, grey; and H, green.

As expected from the chemical composition, the mol­ecular packing is dominated by O—H⋯O and N—H⋯O hydrogen bonding, Table 1 ▸. Each hydroxyl group forms a charge-assisted O—H⋯O hydrogen bond with a carboxyl­ate-O atom; the O1-hydroxyl group also forms a weaker O—H⋯O inter­action with the second carboxyl­ate group. Each of the H1*N*, H2*N* and H3*N* protons of the piperidin-1-ium residues is bifurcated. Two of these inter­actions are intra­molecular N—H⋯O_h_ (h = hydrox­yl) while the remaining N—H⋯O inter­actions, including that formed by the H4*N* atom, have a carboxyl­ate-O atom as the acceptor. The result of the hydrogen bonding is the formation of a supra­molecular chain along the *a* axis, Fig. 3 ▸ *a*. The chains associate *via* C—H⋯F contacts to form the three-dimensional crystal structure, Fig. 3 ▸ *b*; see Table 1 ▸ for parameters describing the closest C—H⋯F contact.

## Database survey   

The crystallographic literature (Groom *et al.*, 2016 ▸) contains at least 16 species related to (I) and a summary of some key geometric descriptors is given in Table 2 ▸. Owing to multiple mol­ecules in several of the structures, *i.e*. two in (II) and (V), three in (VI) and (XV), four in (IV) and five in (III), a reasonable sample of structures is available for comment. The data confirm the proximity of the hy­droxy-O and ammonium-N atoms in these species, and the l-shaped conformation owing to the orthogonal relationship between the quinolinyl and piperidin-1-ium residues. Despite varying compositions in (I)–(XVII), it is apparent that the mol­ecular structures found for mefloquine/mefloquinium cations are robust. Finally, as mentioned above, the phenomenon of kryptoracemates is rare, occurring in just 0.1% of organic crystal structures (Fábián & Brock, 2010 ▸). In this context it might be notable that the structure of (I) is the second example of such behaviour in the structural chemistry of mefloquinium cations, complementing the recent report of (±)-[Mef^+^][O_3_OSC_6_H_4_F-4]Cl (Jotani *et al.*, 2016 ▸).

## Synthesis and crystallization   

Solutions of (±)-erythro-mefloquine base (1 mmol) in EtOH (10 ml) and (+)-PhC(CF_3_)(OMe)CO_2_H (1 mmol) in EtOH (10 ml) were mixed. The reaction mixture was maintained at room temperature and crystals slowly formed over a period of days. Crystals were collected in four batches, at suitable time inter­vals. Only the second batch had crystals suitable for the crystallographic study. The melting points of samples from each batch were similar, in the range 431–436 K. Those used in the X-ray study had m.p. 435–436 K. ^1^H NMR (400 MHz, DMSO-*d*_6_]: δ 1.22–1.25 (4H, *m*), 1.64–1.69 (8H, *m*), 2.95 (4H, *t*, *J* = 11.0 Hz), 3.26 (2H, *brd*, *J* = 11 Hz), 3.48 (2H, *brd*, *J* = 11 Hz), 3.55 (6H, *s*, OMe), 6.05 (2H, *s*), 7.27–7.34 (6H, *m*), 7.74 (4H, *d*, *J* = 7 Hz), 7.80 (1H, *t*, *J* = 8.0 Hz), 7.85 (1H, *t*, *J* = 8.0 Hz), 8.13 (2H, *s*), 8.36 (1H, *d*, *J* = 6.5 Hz), 8.37 (1H, *d*, *J* = 6.8 Hz), 8.85 (1H, *d*, *J* = 8.6 Hz), 8.89 (1H, *d*, *J* = 8.6 Hz). ^13^C NMR (100 MHz, DMSO-*d*_6_]: δ: 21.08, 21.33, 21.72, 44.27,44.33, 54.35, 58.83, 58.87, 67.82, 84.53 (*q*, *J*_CF_ = 23.67 Hz), 115.43, 121.23 (*q*, *J*_CF_ = 273.5 Hz), 123.70 (*q*, *J*_CF_ = 271.7 Hz), 125.03 (*q*, JCF = 286.1 Hz), 127.57, 127.60, 128.07, 128.13, 129.11, 129.86 (*q*, *J*_CF_ = 5 Hz), 136.02, 142.82, 146.73 (*q*, *J*_CF_ = 34.6 Hz), 151.43, 168.18. ^19^F NMR (377 MHz, DMSO-*d*_6_]: δ −58.90 (cation), −66.75 (cation), −69.79 (anion).

## Refinement   

Crystal data, data collection and structure refinement details are summarized in Table 3 ▸. The H atoms were geometrically placed (O—H = 0.84 Å, N—H = 0.92 Å, and C—H = 0.95–1.00 Å) and refined as riding with *U*_iso_(H) = 1.2*U*_eq_(N, C) and 1.5*U*_eq_(O).

## Supplementary Material

Crystal structure: contains datablock(s) I, global. DOI: 10.1107/S2056989016008495/hb7588sup1.cif

Structure factors: contains datablock(s) I. DOI: 10.1107/S2056989016008495/hb7588Isup2.hkl

Supporting information file. DOI: 10.1107/S2056989016008495/hb7588Isup3.cml

CCDC reference: 1481913

Additional supporting information:  crystallographic information; 3D view; checkCIF report

## Figures and Tables

**Table 1:** Hydrogen-bond geometry (Å, °)

*D*—H⋯*A*	*D*—H	H⋯*A*	*D*⋯*A*	*D*—H⋯*A*
O1—H1*O*⋯O3	0.84	1.84	2.615 (3)	153
O1—H1*O*⋯O5	0.84	2.49	3.146 (3)	135
O2—H2*O*⋯O6^i^	0.84	1.92	2.738 (3)	165
N2—H1*N*⋯O1	0.92	2.24	2.677 (3)	108
N2—H1*N*⋯O7^ii^	0.92	2.10	2.817 (3)	134
N2—H2*N*⋯O3^i^	0.92	2.38	3.028 (3)	127
N2—H2*N*⋯O4^i^	0.92	2.03	2.938 (3)	169
N4—H3*N*⋯O2	0.92	2.33	2.734 (3)	106
N4—H3*N*⋯O4^iii^	0.92	2.12	2.849 (3)	136
N4—H4*N*⋯O7	0.92	1.84	2.756 (3)	171
C13—H13⋯F5^i^	1.00	2.38	3.192 (4)	137

Symmetry codes: (i) 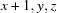; (ii) 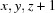; (iii) 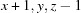.

**Table 2:** Geometric data (Å, °) for mefloquine (Mef) and mefloquinium cations (Mef^+^)

	Formulation	N⋯O	O—C(H)—C(H)—N	(H)C—C—C(OH)—C(H)	REFCODE^*a*^	Ref.
(I)	(±)-[Mef^+^][(+)-PhC(CF_3_)(OMe)CO_2_H]	2.677 (3)–2.734 (3)	−57.5 (4), 59.2 (3)	100.5 (4), −105.1 (4)	–	This work
(II)	(±)-Mef	2.782 (5)–2.846 (5)	−61.2 (4), 66.5 (4)	98.9 (4), −107.2 (4)	LEBYAT	Skórska *et al.* (2006 ▸)
(III)	(−)-Mef	2.754 (4)–2.930 (5)	−58.6 (4) to −71.8 (4)	93.6 (4)–103.8 (4)	QIYREX	Dassonville-Klimpt *et al.* (2013 ▸)
(IV)	(−)-[Mef^+^]Cl^−^·0.25H_2_O	2.722 (15)–2.965 (14)	−54.31 (12) to −71.53 (12)	92.66 (16)–103.52 (14)	BIGTIV	Karle & Karle (2002 ▸)
(V)	(−)-[Mef^+^]Cl^−^·CH_3_OH	2.7052 (18)–2.7792 (16)	54.54 (14), −61.37 (14)	−98.86 (17), 97.92 (17)	SOJPOW01	Pitaluga *et al.* (2010 ▸)
(VI)	(±)-[Mef^+^]Cl^−^·H_2_O	2.720 (3)–2.963 (3)	−56.1 (2), 73.6 (2)	−93.7 (3), 110.86 (24)	HAJSAO	Pitaluga *et al.* (2010 ▸)
(VII)	(±)-[Mef^+^]BPh_4_^−^·CH_3_CH_2_OH	2.701 (3)	−53.0 (2)	98.9 (3)	WAVCED	Wardell *et al.* (2011*a* ▸)
(VIII)	(±)-[Mef^+^][2-NO_2_–C_6_H_4_CO_2_]^−^	2.914 (2)	−72.8 (2)	97.3 (3)	OMELOI	Wardell *et al.* (2011*b* ▸)
(IX)	(±)-[Mef^+^][3-NO_2_–C_6_H_4_CO_2_]^−^	2.7590 (19)	−59.34 (18)	101.00 (21)	OMELUO	Wardell *et al.* (2011*b* ▸)
(XI)	(±)-[Mef^+^][4-NO_2_–C_6_H_4_CO_2_]^−^	2.756 (4)	−54.1 (4)	100.5 (4)	OMEMAV	Wardell *et al.* (2011*b* ▸)
(XI)	(±)-[Mef^+^][3-NH_2_-5-NO_2_–C_6_H_4_CO_2_]^−^·1.5H_2_O	2.867 (3)	66.0 (3)	−102.9 (3)	YAHFIY	de Souza *et al.* (2011 ▸)
(XII)	(±)-[Mef^+^]_2_[CuCl_4_]_2_^−^·4H_2_O	2.886 (5)	−67.4 (4)	103.2 (4)	IHOTAB	Obaleye *et al.* (2009 ▸)
(XIII)	(±)-[Mef^+^]_2_[CdBr_4_]_2_^−^·2CH_3_OH	2.727 (5)	58.6 (5)	−99.6 (6)	IHOTEF	Obaleye *et al.* (2009 ▸)
(XIV)	(±)-[Mef^+^]_3_[CoCl_4_]_2_^−^Cl^−^·H_2_O·CH_3_CH_2_OH	2.710 (4)–3.062 (4)	59.3 (3)–75.2 (3)	−98.9 (4) to −104.3 (3)	LEBYIB	Skórska *et al.* (2006 ▸)
(XV)	(±)-[Mef^+^]_2_[Ph_2_SnCl_4_]^2−^	2.789 (8)	−65.2 (7)	101.0 (8)	PUHVAQ	Wardell *et al.* (2010 ▸)
(XVI)	(±)-[Mef^+^][O_3_OSC_6_H_4_F-4]Cl	2.802 (2)–2.815 (2)	−64.57 (15), 66.50 (14)	−96.69 (17), 94.43 (17)	ELAMAH	Jotani *et al.* (2016 ▸)

Notes: (*a*) Groom *et al.* (2016 ▸).

**Table 3:** Experimental details

Crystal data	
Chemical formula	C_17_H_17_F_6_N_2_O^+^·C_10_H_8_F_3_O_3_^−^
*M*_r_	612.49
Crystal system, space group	Triclinic, *P*1
Temperature (K)	120
*a*, *b*, *c* (Å)	7.5210 (1), 13.3056 (3), 14.8445 (4)
α, β, γ (°)	69.283 (1), 76.336 (2), 85.759 (2)
*V* (Å^3^)	1350.06 (5)
*Z*	2
Radiation type	Mo *K*α
μ (mm^−1^)	0.14
Crystal size (mm)	0.32 × 0.18 × 0.08

Data collection	
Diffractometer	Enraf–Nonius KappaCCD area-detector
Absorption correction	Multi-scan (*SADABS*; Sheldrick, 2007 ▸)
*T*_min_, *T*_max_	0.883, 1.000
No. of measured, independent and observed [*I* > 2σ(*I*)] reflections	28452, 11611, 10550
*R*_int_	0.037
(sin θ/λ)_max_ (Å^−1^)	0.648

Refinement	
*R*[*F* ^2^ > 2σ(*F* ^2^)], *wR*(*F* ^2^), *S*	0.046, 0.108, 1.04
No. of reflections	11611
No. of parameters	761
No. of restraints	3
H-atom treatment	H-atom parameters constrained
Δρ_max_, Δρ_min_ (e Å^−3^)	0.28, −0.30
Absolute structure	Flack *x* determined using 4390 quotients [(*I* ^+^)−(*I* ^−^)]/[(*I* ^+^)+(*I* ^−^)] (Parsons *et al.*, 2013 ▸)
Absolute structure parameter	0.2 (3)

Computer programs: *DENZO* (Otwinowski & Minor, 1997 ▸) and *COLLECT* (Hooft, 1998 ▸), *SHELXS97* (Sheldrick, 2008 ▸), *SHELXL2014* (Sheldrick, 2015 ▸), *ORTEP-3 for Windows* (Farrugia, 2012 ▸), *QMol* (Gans & Shalloway, 2001 ▸), *DIAMOND* (Brandenburg, 2006 ▸) and *publCIF* (Westrip, 2010 ▸).

## supplementary crystallographic information

### Crystal data

**Table:**

C_17_H_17_F_6_N_2_O^+^·C_10_H_8_F_3_O_3_^−^	*Z* = 2
*M**_r_* = 612.49	*F*(000) = 628
Triclinic, *P*1	*D*_x_ = 1.507 Mg m^−^^3^
*a* = 7.5210 (1) Å	Mo *K*α radiation, λ = 0.71073 Å
*b* = 13.3056 (3) Å	Cell parameters from 32656 reflections
*c* = 14.8445 (4) Å	θ = 2.9–27.5°
α = 69.283 (1)°	µ = 0.14 mm^−^^1^
β = 76.336 (2)°	*T* = 120 K
γ = 85.759 (2)°	Prism, colourless
*V* = 1350.06 (5) Å^3^	0.32 × 0.18 × 0.08 mm

### Data collection

**Table:**

Enraf–Nonius KappaCCD area-detector diffractometer	11611 independent reflections
Radiation source: Enraf Nonius FR591 rotating anode	10550 reflections with *I* > 2σ(*I*)
10 cm confocal mirrors monochromator	*R*_int_ = 0.037
Detector resolution: 9.091 pixels mm^-1^	θ_max_ = 27.4°, θ_min_ = 3.0°
φ and ω scans	*h* = −9→9
Absorption correction: multi-scan (*SADABS*; Sheldrick, 2007)	*k* = −17→17
*T*_min_ = 0.883, *T*_max_ = 1.000	*l* = −19→19
28452 measured reflections	

### Refinement

**Table:**

Refinement on *F*^2^	Hydrogen site location: inferred from neighbouring sites
Least-squares matrix: full	H-atom parameters constrained
*R*[*F*^2^ > 2σ(*F*^2^)] = 0.046	*w* = 1/[σ^2^(*F*_o_^2^) + (0.0317*P*)^2^ + 1.2631*P*] where *P* = (*F*_o_^2^ + 2*F*_c_^2^)/3
*wR*(*F*^2^) = 0.108	(Δ/σ)_max_ < 0.001
*S* = 1.04	Δρ_max_ = 0.28 e Å^−^^3^
11611 reflections	Δρ_min_ = −0.30 e Å^−^^3^
761 parameters	Absolute structure: Flack *x* determined using 4390 quotients [(*I*^+^)-(*I*^-^)]/[(*I*^+^)+(*I*^-^)] (Parsons *et al.*, 2013)
3 restraints	Absolute structure parameter: 0.2 (3)

### Special details

**Table:**

Geometry. All esds (except the esd in the dihedral angle between two l.s. planes) are estimated using the full covariance matrix. The cell esds are taken into account individually in the estimation of esds in distances, angles and torsion angles; correlations between esds in cell parameters are only used when they are defined by crystal symmetry. An approximate (isotropic) treatment of cell esds is used for estimating esds involving l.s. planes.

### Fractional atomic coordinates and isotropic or equivalent isotropic displacement parameters (Å^2^)

**Table:**

	*x*	*y*	*z*	*U*_iso_*/*U*_eq_	
F1	0.8444 (4)	0.1170 (3)	0.5300 (2)	0.0497 (8)	
F2	0.5631 (4)	0.1485 (2)	0.5833 (2)	0.0429 (7)	
F3	0.6947 (4)	0.0119 (2)	0.66921 (19)	0.0347 (6)	
F4	0.1398 (4)	0.0877 (2)	0.8274 (2)	0.0398 (6)	
F5	0.0908 (3)	0.1205 (2)	0.9628 (2)	0.0407 (7)	
F6	0.2499 (3)	−0.0157 (2)	0.94808 (19)	0.0312 (6)	
O1	0.4889 (4)	0.3672 (2)	0.9949 (2)	0.0259 (6)	
H1O	0.4170	0.4042	0.9608	0.039*	
N1	0.4983 (5)	0.1398 (3)	0.7841 (2)	0.0225 (7)	
N2	0.8065 (4)	0.3602 (3)	1.0487 (2)	0.0209 (7)	
H1N	0.6967	0.3831	1.0756	0.025*	
H2N	0.8680	0.4185	1.0010	0.025*	
C1	0.3912 (5)	0.1540 (3)	0.8621 (3)	0.0221 (8)	
C2	0.4296 (5)	0.2180 (3)	0.9126 (3)	0.0224 (8)	
H2	0.3435	0.2243	0.9686	0.027*	
C3	0.5949 (5)	0.2716 (3)	0.8793 (3)	0.0206 (8)	
C4	0.7182 (6)	0.2603 (3)	0.7942 (3)	0.0226 (8)	
C5	0.8905 (6)	0.3123 (3)	0.7521 (3)	0.0283 (9)	
H5	0.9307	0.3562	0.7820	0.034*	
C6	1.0004 (7)	0.3003 (4)	0.6688 (3)	0.0344 (10)	
H6	1.1161	0.3357	0.6416	0.041*	
C7	0.9433 (7)	0.2360 (4)	0.6233 (3)	0.0342 (10)	
H7	1.0202	0.2291	0.5650	0.041*	
C8	0.7797 (6)	0.1838 (3)	0.6615 (3)	0.0255 (8)	
C9	0.6618 (6)	0.1942 (3)	0.7480 (3)	0.0234 (8)	
C10	0.7198 (6)	0.1147 (4)	0.6118 (3)	0.0291 (9)	
C11	0.2171 (6)	0.0878 (4)	0.8987 (3)	0.0275 (9)	
C12	0.6438 (5)	0.3415 (3)	0.9326 (3)	0.0202 (7)	
H12	0.7044	0.4093	0.8826	0.024*	
C13	0.7726 (5)	0.2830 (3)	1.0011 (3)	0.0205 (8)	
H13	0.8910	0.2685	0.9602	0.025*	
C14	0.9133 (5)	0.3145 (3)	1.1276 (3)	0.0242 (8)	
H14A	0.9263	0.3690	1.1570	0.029*	
H14B	1.0373	0.2947	1.0984	0.029*	
C15	0.8136 (5)	0.2153 (4)	1.2075 (3)	0.0250 (8)	
H15A	0.8828	0.1852	1.2601	0.030*	
H15B	0.6908	0.2355	1.2377	0.030*	
C16	0.7948 (6)	0.1313 (3)	1.1626 (3)	0.0261 (9)	
H16A	0.7274	0.0679	1.2144	0.031*	
H16B	0.9178	0.1077	1.1364	0.031*	
C17	0.6924 (5)	0.1771 (3)	1.0791 (3)	0.0233 (8)	
H17A	0.6942	0.1235	1.0466	0.028*	
H17B	0.5631	0.1884	1.1081	0.028*	
F7	0.8231 (4)	0.9969 (2)	0.3955 (2)	0.0395 (7)	
F8	0.9419 (5)	0.8584 (3)	0.4868 (2)	0.0489 (8)	
F9	0.6569 (4)	0.8912 (3)	0.5290 (2)	0.0497 (8)	
F10	1.3944 (4)	0.9275 (2)	0.2510 (2)	0.0390 (6)	
F11	1.3021 (3)	1.0328 (2)	0.1238 (2)	0.0348 (6)	
F12	1.4637 (3)	0.8949 (2)	0.1148 (2)	0.0387 (6)	
O2	1.0924 (4)	0.6563 (2)	0.0590 (2)	0.0223 (6)	
H2O	1.1678	0.6251	0.0933	0.033*	
N3	1.0378 (5)	0.8743 (3)	0.2826 (2)	0.0223 (7)	
N4	0.7693 (4)	0.6547 (2)	0.0053 (2)	0.0171 (6)	
H3N	0.8799	0.6369	−0.0258	0.020*	
H4N	0.7169	0.5937	0.0533	0.020*	
C18	1.1552 (5)	0.8637 (3)	0.2051 (3)	0.0220 (8)	
C19	1.1274 (5)	0.8010 (3)	0.1509 (3)	0.0204 (7)	
H19	1.2198	0.7963	0.0965	0.025*	
C20	0.9646 (5)	0.7468 (3)	0.1778 (3)	0.0201 (7)	
C21	0.8330 (6)	0.7544 (3)	0.2612 (3)	0.0209 (8)	
C22	0.6619 (6)	0.6970 (3)	0.2991 (3)	0.0262 (9)	
H22	0.6314	0.6523	0.2671	0.031*	
C23	0.5429 (6)	0.7059 (4)	0.3806 (3)	0.0325 (10)	
H23	0.4301	0.6673	0.4048	0.039*	
C24	0.5843 (6)	0.7714 (4)	0.4295 (3)	0.0308 (9)	
H24	0.4993	0.7768	0.4861	0.037*	
C25	0.7464 (6)	0.8271 (3)	0.3960 (3)	0.0268 (9)	
C26	0.8764 (5)	0.8192 (3)	0.3114 (3)	0.0213 (8)	
C27	0.7921 (7)	0.8934 (4)	0.4511 (3)	0.0315 (9)	
C28	1.3288 (6)	0.9288 (3)	0.1746 (3)	0.0275 (9)	
C29	0.9309 (5)	0.6777 (3)	0.1210 (3)	0.0177 (7)	
H29	0.8751	0.6078	0.1694	0.021*	
C30	0.7994 (5)	0.7324 (3)	0.0531 (3)	0.0191 (7)	
H30	0.6800	0.7448	0.0945	0.023*	
C31	0.6510 (5)	0.6972 (3)	−0.0679 (3)	0.0232 (8)	
H31A	0.5274	0.7127	−0.0343	0.028*	
H31B	0.6384	0.6427	−0.0974	0.028*	
C32	0.7361 (6)	0.7986 (3)	−0.1480 (3)	0.0243 (8)	
H32A	0.6579	0.8269	−0.1965	0.029*	
H32B	0.8576	0.7823	−0.1832	0.029*	
C33	0.7570 (5)	0.8833 (3)	−0.1033 (3)	0.0254 (8)	
H33A	0.6346	0.9033	−0.0723	0.030*	
H33B	0.8163	0.9486	−0.1562	0.030*	
C34	0.8727 (5)	0.8399 (3)	−0.0256 (3)	0.0233 (8)	
H34A	1.0002	0.8306	−0.0588	0.028*	
H34B	0.8744	0.8931	0.0070	0.028*	
F13	0.3672 (4)	0.5765 (2)	0.60704 (18)	0.0363 (6)	
F14	0.0856 (3)	0.5380 (2)	0.68169 (18)	0.0297 (5)	
F15	0.2970 (4)	0.4249 (2)	0.7246 (2)	0.0393 (6)	
O3	0.1897 (4)	0.4383 (3)	0.9334 (2)	0.0442 (9)	
O4	−0.0273 (4)	0.5472 (2)	0.8791 (2)	0.0262 (6)	
O5	0.4526 (3)	0.5488 (2)	0.7955 (2)	0.0242 (6)	
C35	0.1349 (5)	0.5153 (3)	0.8714 (3)	0.0212 (8)	
C36	0.2780 (5)	0.5775 (3)	0.7733 (3)	0.0209 (7)	
C37	0.2558 (5)	0.5295 (3)	0.6963 (3)	0.0259 (8)	
C38	0.6112 (6)	0.6000 (4)	0.7201 (3)	0.0315 (9)	
H38A	0.6417	0.5635	0.6715	0.047*	
H38B	0.7150	0.5957	0.7506	0.047*	
H38C	0.5844	0.6756	0.6870	0.047*	
C39	0.2475 (5)	0.6981 (3)	0.7428 (3)	0.0198 (7)	
C40	0.1796 (6)	0.7609 (3)	0.6613 (3)	0.0258 (8)	
H40	0.1443	0.7283	0.6205	0.031*	
C41	0.1635 (6)	0.8714 (3)	0.6393 (3)	0.0312 (9)	
H41	0.1158	0.9136	0.5840	0.037*	
C42	0.2157 (6)	0.9205 (3)	0.6970 (3)	0.0295 (9)	
H42	0.2072	0.9963	0.6805	0.035*	
C43	0.2807 (6)	0.8583 (3)	0.7790 (3)	0.0280 (9)	
H43	0.3160	0.8912	0.8194	0.034*	
C44	0.2942 (5)	0.7479 (3)	0.8021 (3)	0.0252 (8)	
H44	0.3360	0.7057	0.8595	0.030*	
F16	0.0661 (3)	0.4909 (2)	0.28770 (18)	0.0298 (5)	
F17	0.2010 (3)	0.5936 (2)	0.33665 (19)	0.0315 (6)	
F18	0.1071 (3)	0.4363 (2)	0.43606 (17)	0.0359 (6)	
O6	0.3771 (4)	0.5882 (2)	0.1512 (2)	0.0281 (6)	
O7	0.6151 (4)	0.4788 (2)	0.1645 (2)	0.0225 (6)	
O8	0.4715 (4)	0.4555 (2)	0.3726 (2)	0.0237 (6)	
C45	0.4594 (5)	0.5098 (3)	0.1971 (3)	0.0187 (7)	
C46	0.3690 (5)	0.4435 (3)	0.3076 (3)	0.0180 (7)	
C47	0.1852 (5)	0.4921 (3)	0.3418 (3)	0.0233 (8)	
C48	0.6521 (5)	0.4112 (3)	0.3692 (3)	0.0287 (9)	
H48A	0.6482	0.3364	0.3729	0.043*	
H48B	0.6968	0.4141	0.4252	0.043*	
H48C	0.7346	0.4530	0.3072	0.043*	
C49	0.3369 (5)	0.3262 (3)	0.3213 (3)	0.0198 (7)	
C50	0.3397 (5)	0.2466 (3)	0.4118 (3)	0.0249 (8)	
H50	0.3636	0.2650	0.4641	0.030*	
C51	0.3074 (6)	0.1395 (4)	0.4259 (3)	0.0337 (10)	
H51	0.3121	0.0850	0.4874	0.040*	
C52	0.2689 (6)	0.1130 (4)	0.3511 (4)	0.0347 (10)	
H52	0.2460	0.0402	0.3609	0.042*	
C53	0.2636 (5)	0.1926 (3)	0.2613 (3)	0.0286 (9)	
H53	0.2352	0.1743	0.2099	0.034*	
C54	0.2994 (5)	0.2989 (3)	0.2460 (3)	0.0233 (8)	
H54	0.2983	0.3530	0.1838	0.028*	

### Atomic displacement parameters (Å^2^)

**Table:**

	*U*^11^	*U*^22^	*U*^33^	*U*^12^	*U*^13^	*U*^23^
F1	0.0631 (19)	0.060 (2)	0.0295 (14)	−0.0243 (15)	0.0129 (13)	−0.0303 (14)
F2	0.0589 (19)	0.0427 (17)	0.0374 (15)	0.0072 (13)	−0.0276 (14)	−0.0170 (13)
F3	0.0508 (16)	0.0244 (13)	0.0304 (14)	−0.0034 (11)	−0.0084 (11)	−0.0109 (11)
F4	0.0317 (14)	0.0498 (17)	0.0427 (15)	−0.0023 (12)	−0.0147 (12)	−0.0171 (13)
F5	0.0255 (13)	0.0466 (17)	0.0549 (18)	0.0005 (11)	0.0042 (12)	−0.0319 (14)
F6	0.0330 (13)	0.0245 (13)	0.0332 (13)	−0.0046 (10)	−0.0015 (11)	−0.0095 (11)
O1	0.0275 (15)	0.0279 (15)	0.0245 (14)	0.0096 (12)	−0.0075 (11)	−0.0125 (12)
N1	0.0262 (17)	0.0194 (17)	0.0207 (16)	0.0009 (13)	−0.0049 (13)	−0.0059 (13)
N2	0.0208 (16)	0.0209 (16)	0.0202 (16)	−0.0011 (12)	−0.0016 (12)	−0.0081 (13)
C1	0.0209 (18)	0.0196 (19)	0.0230 (19)	0.0046 (15)	−0.0048 (15)	−0.0049 (16)
C2	0.028 (2)	0.0185 (19)	0.0189 (18)	0.0018 (15)	−0.0024 (15)	−0.0068 (15)
C3	0.028 (2)	0.0154 (18)	0.0167 (17)	0.0025 (15)	−0.0049 (15)	−0.0040 (14)
C4	0.029 (2)	0.0205 (19)	0.0156 (17)	0.0002 (16)	−0.0027 (15)	−0.0049 (15)
C5	0.041 (2)	0.024 (2)	0.0193 (19)	−0.0124 (18)	0.0000 (17)	−0.0089 (17)
C6	0.040 (2)	0.034 (2)	0.024 (2)	−0.016 (2)	0.0081 (18)	−0.0111 (19)
C7	0.044 (3)	0.034 (2)	0.021 (2)	−0.009 (2)	0.0060 (18)	−0.0122 (18)
C8	0.037 (2)	0.020 (2)	0.0177 (18)	−0.0026 (16)	−0.0030 (16)	−0.0056 (16)
C9	0.031 (2)	0.0189 (19)	0.0178 (18)	−0.0025 (15)	−0.0052 (15)	−0.0028 (15)
C10	0.039 (2)	0.030 (2)	0.0189 (19)	−0.0019 (18)	−0.0024 (16)	−0.0118 (17)
C11	0.0216 (19)	0.032 (2)	0.030 (2)	0.0035 (16)	−0.0051 (16)	−0.0132 (18)
C12	0.0243 (19)	0.0206 (19)	0.0167 (17)	0.0028 (14)	−0.0037 (14)	−0.0086 (15)
C13	0.0235 (19)	0.0157 (18)	0.0216 (18)	0.0025 (14)	−0.0021 (15)	−0.0079 (15)
C14	0.0209 (18)	0.027 (2)	0.0226 (19)	−0.0002 (15)	−0.0042 (15)	−0.0057 (16)
C15	0.0210 (19)	0.031 (2)	0.0189 (19)	−0.0018 (16)	−0.0040 (15)	−0.0031 (17)
C16	0.029 (2)	0.022 (2)	0.022 (2)	0.0024 (16)	−0.0057 (16)	−0.0011 (16)
C17	0.026 (2)	0.0181 (19)	0.0214 (19)	−0.0001 (15)	−0.0039 (15)	−0.0025 (15)
F7	0.0598 (18)	0.0283 (14)	0.0327 (14)	−0.0084 (12)	−0.0043 (12)	−0.0156 (12)
F8	0.066 (2)	0.0526 (19)	0.0418 (17)	0.0007 (15)	−0.0319 (15)	−0.0207 (15)
F9	0.0633 (19)	0.059 (2)	0.0316 (15)	−0.0221 (15)	0.0105 (13)	−0.0306 (14)
F10	0.0332 (14)	0.0489 (17)	0.0464 (16)	−0.0057 (12)	−0.0172 (12)	−0.0237 (14)
F11	0.0332 (14)	0.0248 (13)	0.0459 (16)	−0.0073 (10)	−0.0053 (11)	−0.0125 (12)
F12	0.0227 (12)	0.0434 (16)	0.0578 (18)	−0.0033 (11)	0.0000 (11)	−0.0321 (14)
O2	0.0219 (13)	0.0269 (14)	0.0220 (13)	0.0088 (11)	−0.0088 (11)	−0.0124 (11)
N3	0.0274 (17)	0.0194 (17)	0.0217 (16)	0.0006 (13)	−0.0075 (13)	−0.0078 (13)
N4	0.0180 (14)	0.0178 (15)	0.0141 (14)	−0.0018 (11)	−0.0026 (11)	−0.0042 (12)
C18	0.0221 (19)	0.0191 (19)	0.027 (2)	0.0016 (15)	−0.0084 (15)	−0.0084 (16)
C19	0.0223 (18)	0.0192 (18)	0.0188 (17)	0.0016 (14)	−0.0061 (14)	−0.0048 (15)
C20	0.0256 (19)	0.0169 (18)	0.0192 (18)	0.0005 (14)	−0.0095 (15)	−0.0053 (15)
C21	0.030 (2)	0.0148 (18)	0.0160 (17)	−0.0031 (15)	−0.0058 (15)	−0.0023 (14)
C22	0.031 (2)	0.025 (2)	0.023 (2)	−0.0087 (16)	−0.0051 (16)	−0.0082 (17)
C23	0.035 (2)	0.036 (2)	0.024 (2)	−0.0168 (19)	0.0047 (17)	−0.0120 (19)
C24	0.038 (2)	0.033 (2)	0.021 (2)	−0.0094 (19)	0.0012 (17)	−0.0108 (18)
C25	0.037 (2)	0.026 (2)	0.0181 (19)	−0.0039 (17)	−0.0057 (16)	−0.0079 (16)
C26	0.0267 (19)	0.0197 (19)	0.0176 (18)	−0.0021 (15)	−0.0066 (14)	−0.0050 (15)
C27	0.042 (2)	0.029 (2)	0.022 (2)	−0.0085 (18)	−0.0026 (18)	−0.0087 (17)
C28	0.028 (2)	0.024 (2)	0.037 (2)	0.0018 (16)	−0.0083 (17)	−0.0176 (18)
C29	0.0212 (18)	0.0144 (17)	0.0184 (17)	0.0000 (14)	−0.0062 (14)	−0.0055 (14)
C30	0.0190 (18)	0.0209 (19)	0.0195 (17)	0.0020 (14)	−0.0057 (14)	−0.0092 (15)
C31	0.0212 (19)	0.031 (2)	0.0175 (18)	−0.0010 (16)	−0.0081 (15)	−0.0063 (16)
C32	0.0236 (19)	0.028 (2)	0.0178 (18)	−0.0015 (16)	−0.0054 (15)	−0.0028 (16)
C33	0.0240 (19)	0.024 (2)	0.0215 (19)	0.0012 (16)	−0.0058 (15)	−0.0002 (16)
C34	0.0237 (19)	0.020 (2)	0.027 (2)	−0.0011 (15)	−0.0075 (15)	−0.0076 (16)
F13	0.0380 (14)	0.0484 (16)	0.0237 (12)	−0.0084 (12)	0.0015 (10)	−0.0176 (11)
F14	0.0332 (13)	0.0310 (13)	0.0282 (12)	−0.0065 (10)	−0.0116 (10)	−0.0097 (10)
F15	0.0560 (17)	0.0280 (14)	0.0418 (15)	0.0100 (12)	−0.0183 (13)	−0.0188 (12)
O3	0.0226 (15)	0.046 (2)	0.0366 (18)	0.0038 (14)	0.0019 (13)	0.0131 (15)
O4	0.0175 (13)	0.0333 (16)	0.0263 (14)	−0.0003 (11)	−0.0037 (11)	−0.0090 (12)
O5	0.0162 (12)	0.0272 (14)	0.0226 (13)	−0.0004 (10)	−0.0016 (10)	−0.0024 (11)
C35	0.0208 (18)	0.0204 (18)	0.0208 (18)	−0.0023 (14)	−0.0025 (14)	−0.0060 (15)
C36	0.0176 (17)	0.0231 (18)	0.0213 (18)	−0.0012 (14)	−0.0057 (14)	−0.0057 (15)
C37	0.028 (2)	0.025 (2)	0.0231 (19)	−0.0034 (16)	−0.0025 (15)	−0.0083 (16)
C38	0.0215 (19)	0.037 (2)	0.028 (2)	−0.0034 (17)	0.0017 (16)	−0.0065 (18)
C39	0.0180 (17)	0.0206 (18)	0.0185 (17)	−0.0015 (14)	−0.0004 (13)	−0.0063 (14)
C40	0.031 (2)	0.025 (2)	0.0228 (19)	−0.0068 (16)	−0.0083 (16)	−0.0071 (16)
C41	0.040 (2)	0.026 (2)	0.025 (2)	−0.0070 (18)	−0.0129 (18)	−0.0009 (17)
C42	0.035 (2)	0.021 (2)	0.031 (2)	−0.0054 (17)	−0.0048 (17)	−0.0072 (17)
C43	0.027 (2)	0.031 (2)	0.032 (2)	−0.0022 (17)	−0.0064 (17)	−0.0185 (18)
C44	0.0236 (19)	0.033 (2)	0.0211 (18)	0.0030 (16)	−0.0083 (15)	−0.0103 (16)
F16	0.0173 (11)	0.0350 (14)	0.0323 (13)	−0.0005 (9)	−0.0058 (9)	−0.0057 (11)
F17	0.0366 (13)	0.0262 (13)	0.0322 (13)	0.0084 (10)	−0.0066 (11)	−0.0130 (11)
F18	0.0326 (13)	0.0398 (15)	0.0226 (12)	0.0048 (11)	0.0052 (10)	−0.0037 (11)
O6	0.0230 (14)	0.0281 (15)	0.0228 (14)	0.0025 (11)	−0.0030 (11)	0.0017 (12)
O7	0.0203 (13)	0.0222 (13)	0.0209 (13)	0.0011 (10)	−0.0006 (10)	−0.0053 (11)
O8	0.0248 (14)	0.0253 (14)	0.0221 (14)	−0.0017 (11)	−0.0094 (11)	−0.0067 (11)
C45	0.0172 (17)	0.0194 (18)	0.0187 (17)	−0.0039 (14)	−0.0025 (13)	−0.0059 (14)
C46	0.0171 (16)	0.0201 (17)	0.0160 (16)	−0.0037 (13)	−0.0022 (13)	−0.0056 (13)
C47	0.0229 (19)	0.0233 (19)	0.0216 (18)	0.0007 (15)	−0.0013 (14)	−0.0078 (15)
C48	0.0215 (19)	0.035 (2)	0.028 (2)	−0.0048 (16)	−0.0109 (16)	−0.0046 (17)
C49	0.0129 (16)	0.0214 (18)	0.0220 (18)	−0.0045 (14)	0.0005 (14)	−0.0056 (15)
C50	0.025 (2)	0.026 (2)	0.0219 (19)	−0.0046 (16)	−0.0067 (15)	−0.0053 (16)
C51	0.041 (2)	0.023 (2)	0.030 (2)	−0.0042 (18)	−0.0041 (18)	−0.0015 (17)
C52	0.031 (2)	0.028 (2)	0.043 (3)	−0.0096 (18)	0.0013 (19)	−0.014 (2)
C53	0.0204 (19)	0.031 (2)	0.037 (2)	−0.0031 (16)	−0.0038 (16)	−0.0173 (19)
C54	0.0208 (18)	0.027 (2)	0.0198 (18)	−0.0033 (15)	−0.0024 (14)	−0.0067 (16)

### Geometric parameters (Å, º)

**Table:**

F1—C10	1.339 (5)	C23—C24	1.406 (6)
F2—C10	1.339 (5)	C23—H23	0.9500
F3—C10	1.330 (5)	C24—C25	1.368 (6)
F4—C11	1.323 (5)	C24—H24	0.9500
F5—C11	1.343 (5)	C25—C26	1.429 (6)
F6—C11	1.346 (5)	C25—C27	1.502 (6)
O1—C12	1.410 (4)	C29—C30	1.539 (5)
O1—H1O	0.8400	C29—H29	1.0000
N1—C1	1.310 (5)	C30—C34	1.531 (5)
N1—C9	1.367 (5)	C30—H30	1.0000
N2—C13	1.502 (5)	C31—C32	1.509 (6)
N2—C14	1.502 (5)	C31—H31A	0.9900
N2—H1N	0.9100	C31—H31B	0.9900
N2—H2N	0.9100	C32—C33	1.530 (6)
C1—C2	1.401 (6)	C32—H32A	0.9900
C1—C11	1.510 (6)	C32—H32B	0.9900
C2—C3	1.374 (6)	C33—C34	1.534 (5)
C2—H2	0.9500	C33—H33A	0.9900
C3—C4	1.427 (5)	C33—H33B	0.9900
C3—C12	1.528 (5)	C34—H34A	0.9900
C4—C5	1.411 (6)	C34—H34B	0.9900
C4—C9	1.429 (5)	F13—C37	1.347 (5)
C5—C6	1.369 (6)	F14—C37	1.339 (5)
C5—H5	0.9500	F15—C37	1.339 (5)
C6—C7	1.405 (6)	O3—C35	1.231 (5)
C6—H6	0.9500	O4—C35	1.255 (5)
C7—C8	1.356 (6)	O5—C36	1.420 (4)
C7—H7	0.9500	O5—C38	1.446 (5)
C8—C9	1.423 (5)	C35—C36	1.582 (5)
C8—C10	1.514 (6)	C36—C39	1.521 (5)
C12—C13	1.540 (5)	C36—C37	1.537 (5)
C12—H12	1.0000	C38—H38A	0.9800
C13—C17	1.527 (5)	C38—H38B	0.9800
C13—H13	1.0000	C38—H38C	0.9800
C14—C15	1.527 (6)	C39—C40	1.392 (6)
C14—H14A	0.9900	C39—C44	1.389 (5)
C14—H14B	0.9900	C40—C41	1.391 (6)
C15—C16	1.518 (6)	C40—H40	0.9500
C15—H15A	0.9900	C41—C42	1.381 (6)
C15—H15B	0.9900	C41—H41	0.9500
C16—C17	1.532 (5)	C42—C43	1.383 (6)
C16—H16A	0.9900	C42—H42	0.9500
C16—H16B	0.9900	C43—C44	1.387 (6)
C17—H17A	0.9900	C43—H43	0.9500
C17—H17B	0.9900	C44—H44	0.9500
F7—C27	1.335 (5)	F16—C47	1.341 (4)
F8—C27	1.340 (6)	F17—C47	1.338 (5)
F9—C27	1.340 (5)	F18—C47	1.340 (4)
F10—C28	1.332 (5)	O6—C45	1.239 (5)
F11—C28	1.347 (5)	O7—C45	1.257 (4)
F12—C28	1.346 (5)	O8—C46	1.425 (4)
O2—C29	1.417 (4)	O8—C48	1.437 (5)
O2—H2O	0.8400	C45—C46	1.571 (5)
N3—C18	1.318 (5)	C46—C49	1.531 (5)
N3—C26	1.363 (5)	C46—C47	1.535 (5)
N4—C31	1.498 (5)	C48—H48A	0.9800
N4—C30	1.500 (5)	C48—H48B	0.9800
N4—H3N	0.9100	C48—H48C	0.9800
N4—H4N	0.9100	C49—C54	1.383 (5)
C18—C19	1.403 (5)	C49—C50	1.389 (5)
C18—C28	1.507 (6)	C50—C51	1.395 (6)
C19—C20	1.370 (5)	C50—H50	0.9500
C19—H19	0.9500	C51—C52	1.374 (7)
C20—C21	1.421 (5)	C51—H51	0.9500
C20—C29	1.518 (5)	C52—C53	1.386 (7)
C21—C26	1.420 (5)	C52—H52	0.9500
C21—C22	1.433 (5)	C53—C54	1.388 (6)
C22—C23	1.361 (6)	C53—H53	0.9500
C22—H22	0.9500	C54—H54	0.9500
			
C12—O1—H1O	109.5	F9—C27—C25	111.9 (4)
C1—N1—C9	116.6 (3)	F10—C28—F11	106.5 (3)
C13—N2—C14	114.5 (3)	F10—C28—F12	106.9 (4)
C13—N2—H1N	108.6	F11—C28—F12	106.1 (4)
C14—N2—H1N	108.6	F10—C28—C18	113.3 (4)
C13—N2—H2N	108.6	F11—C28—C18	111.2 (3)
C14—N2—H2N	108.6	F12—C28—C18	112.4 (3)
H1N—N2—H2N	107.6	O2—C29—C20	113.2 (3)
N1—C1—C2	126.2 (4)	O2—C29—C30	106.1 (3)
N1—C1—C11	112.8 (4)	C20—C29—C30	111.4 (3)
C2—C1—C11	120.9 (3)	O2—C29—H29	108.7
C3—C2—C1	118.3 (4)	C20—C29—H29	108.7
C3—C2—H2	120.8	C30—C29—H29	108.7
C1—C2—H2	120.8	N4—C30—C34	110.5 (3)
C2—C3—C4	118.5 (4)	N4—C30—C29	106.5 (3)
C2—C3—C12	120.3 (3)	C34—C30—C29	113.2 (3)
C4—C3—C12	121.2 (3)	N4—C30—H30	108.9
C5—C4—C9	118.3 (3)	C34—C30—H30	108.9
C5—C4—C3	123.8 (4)	C29—C30—H30	108.9
C9—C4—C3	117.9 (4)	N4—C31—C32	109.5 (3)
C6—C5—C4	120.9 (4)	N4—C31—H31A	109.8
C6—C5—H5	119.6	C32—C31—H31A	109.8
C4—C5—H5	119.6	N4—C31—H31B	109.8
C5—C6—C7	120.5 (4)	C32—C31—H31B	109.8
C5—C6—H6	119.7	H31A—C31—H31B	108.2
C7—C6—H6	119.7	C31—C32—C33	110.3 (3)
C8—C7—C6	120.7 (4)	C31—C32—H32A	109.6
C8—C7—H7	119.6	C33—C32—H32A	109.6
C6—C7—H7	119.6	C31—C32—H32B	109.6
C7—C8—C9	120.4 (4)	C33—C32—H32B	109.6
C7—C8—C10	120.2 (4)	H32A—C32—H32B	108.1
C9—C8—C10	119.4 (4)	C32—C33—C34	110.5 (3)
N1—C9—C8	118.4 (4)	C32—C33—H33A	109.5
N1—C9—C4	122.4 (3)	C34—C33—H33A	109.5
C8—C9—C4	119.2 (4)	C32—C33—H33B	109.5
F3—C10—F1	106.6 (4)	C34—C33—H33B	109.5
F3—C10—F2	107.0 (4)	H33A—C33—H33B	108.1
F1—C10—F2	106.3 (4)	C30—C34—C33	112.3 (3)
F3—C10—C8	113.2 (3)	C30—C34—H34A	109.1
F1—C10—C8	111.2 (4)	C33—C34—H34A	109.1
F2—C10—C8	112.2 (4)	C30—C34—H34B	109.1
F4—C11—F5	107.4 (3)	C33—C34—H34B	109.1
F4—C11—F6	106.5 (4)	H34A—C34—H34B	107.9
F5—C11—F6	105.7 (4)	C36—O5—C38	117.4 (3)
F4—C11—C1	113.8 (4)	O3—C35—O4	125.0 (4)
F5—C11—C1	112.3 (4)	O3—C35—C36	117.9 (3)
F6—C11—C1	110.7 (3)	O4—C35—C36	117.1 (3)
O1—C12—C3	112.2 (3)	O5—C36—C39	112.1 (3)
O1—C12—C13	105.0 (3)	O5—C36—C37	108.6 (3)
C3—C12—C13	111.7 (3)	C39—C36—C37	113.8 (3)
O1—C12—H12	109.3	O5—C36—C35	105.3 (3)
C3—C12—H12	109.3	C39—C36—C35	110.9 (3)
C13—C12—H12	109.3	C37—C36—C35	105.7 (3)
N2—C13—C17	110.9 (3)	F14—C37—F15	107.1 (3)
N2—C13—C12	105.6 (3)	F14—C37—F13	106.4 (3)
C17—C13—C12	113.0 (3)	F15—C37—F13	106.3 (3)
N2—C13—H13	109.1	F14—C37—C36	113.1 (3)
C17—C13—H13	109.1	F15—C37—C36	110.7 (3)
C12—C13—H13	109.1	F13—C37—C36	112.8 (3)
N2—C14—C15	109.3 (3)	O5—C38—H38A	109.5
N2—C14—H14A	109.8	O5—C38—H38B	109.5
C15—C14—H14A	109.8	H38A—C38—H38B	109.5
N2—C14—H14B	109.8	O5—C38—H38C	109.5
C15—C14—H14B	109.8	H38A—C38—H38C	109.5
H14A—C14—H14B	108.3	H38B—C38—H38C	109.5
C16—C15—C14	109.8 (3)	C40—C39—C44	118.6 (4)
C16—C15—H15A	109.7	C40—C39—C36	125.5 (3)
C14—C15—H15A	109.7	C44—C39—C36	115.9 (3)
C16—C15—H15B	109.7	C41—C40—C39	120.0 (4)
C14—C15—H15B	109.7	C41—C40—H40	120.0
H15A—C15—H15B	108.2	C39—C40—H40	120.0
C15—C16—C17	110.7 (3)	C40—C41—C42	120.9 (4)
C15—C16—H16A	109.5	C40—C41—H41	119.5
C17—C16—H16A	109.5	C42—C41—H41	119.5
C15—C16—H16B	109.5	C43—C42—C41	119.3 (4)
C17—C16—H16B	109.5	C43—C42—H42	120.4
H16A—C16—H16B	108.1	C41—C42—H42	120.4
C13—C17—C16	113.8 (3)	C42—C43—C44	120.1 (4)
C13—C17—H17A	108.8	C42—C43—H43	120.0
C16—C17—H17A	108.8	C44—C43—H43	120.0
C13—C17—H17B	108.8	C43—C44—C39	121.1 (4)
C16—C17—H17B	108.8	C43—C44—H44	119.4
H17A—C17—H17B	107.7	C39—C44—H44	119.4
C29—O2—H2O	109.5	C46—O8—C48	118.7 (3)
C18—N3—C26	116.5 (3)	O6—C45—O7	125.9 (3)
C31—N4—C30	113.9 (3)	O6—C45—C46	118.9 (3)
C31—N4—H3N	108.8	O7—C45—C46	115.1 (3)
C30—N4—H3N	108.8	O8—C46—C49	113.5 (3)
C31—N4—H4N	108.8	O8—C46—C47	101.2 (3)
C30—N4—H4N	108.8	C49—C46—C47	108.7 (3)
H3N—N4—H4N	107.7	O8—C46—C45	110.8 (3)
N3—C18—C19	125.5 (4)	C49—C46—C45	111.4 (3)
N3—C18—C28	114.2 (3)	C47—C46—C45	110.9 (3)
C19—C18—C28	120.2 (4)	F16—C47—F17	107.7 (3)
C20—C19—C18	118.7 (4)	F16—C47—F18	106.7 (3)
C20—C19—H19	120.6	F17—C47—F18	106.4 (3)
C18—C19—H19	120.6	F16—C47—C46	111.2 (3)
C19—C20—C21	118.3 (3)	F17—C47—C46	113.0 (3)
C19—C20—C29	120.0 (3)	F18—C47—C46	111.4 (3)
C21—C20—C29	121.6 (3)	O8—C48—H48A	109.5
C20—C21—C26	118.2 (3)	O8—C48—H48B	109.5
C20—C21—C22	123.3 (3)	H48A—C48—H48B	109.5
C26—C21—C22	118.6 (3)	O8—C48—H48C	109.5
C23—C22—C21	120.5 (4)	H48A—C48—H48C	109.5
C23—C22—H22	119.7	H48B—C48—H48C	109.5
C21—C22—H22	119.7	C54—C49—C50	119.7 (4)
C22—C23—C24	121.1 (4)	C54—C49—C46	120.8 (3)
C22—C23—H23	119.5	C50—C49—C46	119.5 (3)
C24—C23—H23	119.5	C49—C50—C51	120.1 (4)
C25—C24—C23	120.3 (4)	C49—C50—H50	119.9
C25—C24—H24	119.9	C51—C50—H50	119.9
C23—C24—H24	119.9	C52—C51—C50	120.0 (4)
C24—C25—C26	120.6 (4)	C52—C51—H51	120.0
C24—C25—C27	119.7 (4)	C50—C51—H51	120.0
C26—C25—C27	119.5 (4)	C51—C52—C53	119.9 (4)
N3—C26—C21	122.8 (3)	C51—C52—H52	120.1
N3—C26—C25	118.3 (3)	C53—C52—H52	120.1
C21—C26—C25	119.0 (4)	C52—C53—C54	120.4 (4)
F7—C27—F8	106.4 (4)	C52—C53—H53	119.8
F7—C27—F9	106.4 (4)	C54—C53—H53	119.8
F8—C27—F9	106.3 (4)	C49—C54—C53	119.9 (4)
F7—C27—C25	113.1 (4)	C49—C54—H54	120.0
F8—C27—C25	112.3 (4)	C53—C54—H54	120.0
			
C9—N1—C1—C2	−1.2 (6)	N3—C18—C28—F10	41.5 (5)
C9—N1—C1—C11	−178.0 (3)	C19—C18—C28—F10	−140.0 (4)
N1—C1—C2—C3	−0.3 (6)	N3—C18—C28—F11	−78.4 (5)
C11—C1—C2—C3	176.2 (4)	C19—C18—C28—F11	100.1 (4)
C1—C2—C3—C4	0.7 (6)	N3—C18—C28—F12	162.9 (4)
C1—C2—C3—C12	−179.4 (3)	C19—C18—C28—F12	−18.7 (6)
C2—C3—C4—C5	179.1 (4)	C19—C20—C29—O2	14.4 (5)
C12—C3—C4—C5	−0.9 (6)	C21—C20—C29—O2	−163.5 (3)
C2—C3—C4—C9	0.5 (6)	C19—C20—C29—C30	−105.1 (4)
C12—C3—C4—C9	−179.5 (4)	C21—C20—C29—C30	77.0 (4)
C9—C4—C5—C6	0.3 (7)	C31—N4—C30—C34	−53.8 (4)
C3—C4—C5—C6	−178.2 (4)	C31—N4—C30—C29	−177.1 (3)
C4—C5—C6—C7	0.2 (8)	O2—C29—C30—N4	59.2 (3)
C5—C6—C7—C8	−0.8 (8)	C20—C29—C30—N4	−177.2 (3)
C6—C7—C8—C9	0.7 (7)	O2—C29—C30—C34	−62.4 (4)
C6—C7—C8—C10	−180.0 (4)	C20—C29—C30—C34	61.3 (4)
C1—N1—C9—C8	−178.0 (4)	C30—N4—C31—C32	58.5 (4)
C1—N1—C9—C4	2.4 (6)	N4—C31—C32—C33	−59.2 (4)
C7—C8—C9—N1	−179.8 (4)	C31—C32—C33—C34	57.5 (4)
C10—C8—C9—N1	0.9 (6)	N4—C30—C34—C33	50.6 (4)
C7—C8—C9—C4	−0.2 (6)	C29—C30—C34—C33	169.9 (3)
C10—C8—C9—C4	−179.5 (4)	C32—C33—C34—C30	−53.4 (4)
C5—C4—C9—N1	179.2 (4)	C38—O5—C36—C39	−55.4 (4)
C3—C4—C9—N1	−2.1 (6)	C38—O5—C36—C37	71.1 (4)
C5—C4—C9—C8	−0.4 (6)	C38—O5—C36—C35	−176.1 (3)
C3—C4—C9—C8	178.3 (4)	O3—C35—C36—O5	−18.6 (5)
C7—C8—C10—F3	118.6 (5)	O4—C35—C36—O5	162.9 (3)
C9—C8—C10—F3	−62.1 (5)	O3—C35—C36—C39	−140.0 (4)
C7—C8—C10—F1	−1.4 (6)	O4—C35—C36—C39	41.4 (5)
C9—C8—C10—F1	177.9 (4)	O3—C35—C36—C37	96.2 (4)
C7—C8—C10—F2	−120.2 (5)	O4—C35—C36—C37	−82.3 (4)
C9—C8—C10—F2	59.1 (5)	O5—C36—C37—F14	170.4 (3)
N1—C1—C11—F4	−43.3 (5)	C39—C36—C37—F14	−64.1 (4)
C2—C1—C11—F4	139.7 (4)	C35—C36—C37—F14	57.8 (4)
N1—C1—C11—F5	−165.5 (4)	O5—C36—C37—F15	50.1 (4)
C2—C1—C11—F5	17.5 (5)	C39—C36—C37—F15	175.7 (3)
N1—C1—C11—F6	76.6 (4)	C35—C36—C37—F15	−62.4 (4)
C2—C1—C11—F6	−100.4 (4)	O5—C36—C37—F13	−68.8 (4)
C2—C3—C12—O1	−17.1 (5)	C39—C36—C37—F13	56.7 (4)
C4—C3—C12—O1	162.9 (3)	C35—C36—C37—F13	178.6 (3)
C2—C3—C12—C13	100.5 (4)	O5—C36—C39—C40	132.9 (4)
C4—C3—C12—C13	−79.6 (4)	C37—C36—C39—C40	9.2 (5)
C14—N2—C13—C17	50.8 (4)	C35—C36—C39—C40	−109.8 (4)
C14—N2—C13—C12	173.6 (3)	O5—C36—C39—C44	−46.5 (4)
O1—C12—C13—N2	−57.5 (4)	C37—C36—C39—C44	−170.1 (3)
C3—C12—C13—N2	−179.4 (3)	C35—C36—C39—C44	70.9 (4)
O1—C12—C13—C17	63.8 (4)	C44—C39—C40—C41	1.4 (6)
C3—C12—C13—C17	−58.0 (4)	C36—C39—C40—C41	−177.9 (4)
C13—N2—C14—C15	−57.5 (4)	C39—C40—C41—C42	0.7 (6)
N2—C14—C15—C16	59.8 (4)	C40—C41—C42—C43	−1.6 (7)
C14—C15—C16—C17	−57.8 (4)	C41—C42—C43—C44	0.5 (6)
N2—C13—C17—C16	−47.7 (4)	C42—C43—C44—C39	1.6 (6)
C12—C13—C17—C16	−166.0 (3)	C40—C39—C44—C43	−2.5 (6)
C15—C16—C17—C13	52.4 (4)	C36—C39—C44—C43	176.9 (4)
C26—N3—C18—C19	−0.1 (6)	C48—O8—C46—C49	59.0 (4)
C26—N3—C18—C28	178.3 (4)	C48—O8—C46—C47	175.3 (3)
N3—C18—C19—C20	1.3 (6)	C48—O8—C46—C45	−67.1 (4)
C28—C18—C19—C20	−177.0 (4)	O6—C45—C46—O8	−112.9 (4)
C18—C19—C20—C21	−1.6 (6)	O7—C45—C46—O8	65.1 (4)
C18—C19—C20—C29	−179.6 (3)	O6—C45—C46—C49	119.8 (4)
C19—C20—C21—C26	1.0 (5)	O7—C45—C46—C49	−62.2 (4)
C29—C20—C21—C26	178.9 (3)	O6—C45—C46—C47	−1.4 (5)
C19—C20—C21—C22	−176.8 (4)	O7—C45—C46—C47	176.5 (3)
C29—C20—C21—C22	1.1 (6)	O8—C46—C47—F16	178.8 (3)
C20—C21—C22—C23	178.7 (4)	C49—C46—C47—F16	−61.5 (4)
C26—C21—C22—C23	1.0 (6)	C45—C46—C47—F16	61.3 (4)
C21—C22—C23—C24	0.0 (7)	O8—C46—C47—F17	57.4 (4)
C22—C23—C24—C25	−0.3 (8)	C49—C46—C47—F17	177.2 (3)
C23—C24—C25—C26	−0.4 (7)	C45—C46—C47—F17	−60.1 (4)
C23—C24—C25—C27	−177.7 (4)	O8—C46—C47—F18	−62.3 (4)
C18—N3—C26—C21	−0.7 (6)	C49—C46—C47—F18	57.4 (4)
C18—N3—C26—C25	179.0 (4)	C45—C46—C47—F18	−179.8 (3)
C20—C21—C26—N3	0.2 (6)	O8—C46—C49—C54	−157.3 (3)
C22—C21—C26—N3	178.1 (4)	C47—C46—C49—C54	91.0 (4)
C20—C21—C26—C25	−179.5 (4)	C45—C46—C49—C54	−31.4 (5)
C22—C21—C26—C25	−1.6 (6)	O8—C46—C49—C50	24.8 (5)
C24—C25—C26—N3	−178.3 (4)	C47—C46—C49—C50	−86.9 (4)
C27—C25—C26—N3	−1.1 (6)	C45—C46—C49—C50	150.6 (3)
C24—C25—C26—C21	1.4 (6)	C54—C49—C50—C51	0.9 (6)
C27—C25—C26—C21	178.6 (4)	C46—C49—C50—C51	178.8 (4)
C24—C25—C27—F7	−122.0 (5)	C49—C50—C51—C52	−1.4 (7)
C26—C25—C27—F7	60.7 (6)	C50—C51—C52—C53	0.5 (7)
C24—C25—C27—F8	117.5 (5)	C51—C52—C53—C54	0.9 (6)
C26—C25—C27—F8	−59.8 (5)	C50—C49—C54—C53	0.5 (6)
C24—C25—C27—F9	−1.9 (6)	C46—C49—C54—C53	−177.4 (3)
C26—C25—C27—F9	−179.2 (4)	C52—C53—C54—C49	−1.4 (6)

### Hydrogen-bond geometry (Å, º)

**Table:**

*D*—H···*A*	*D*—H	H···*A*	*D*···*A*	*D*—H···*A*
O1—H1*O*···O3	0.84	1.84	2.615 (3)	153
O1—H1*O*···O5	0.84	2.49	3.146 (3)	135
O2—H2*O*···O6^i^	0.84	1.92	2.738 (3)	165
N2—H1*N*···O1	0.92	2.24	2.677 (3)	108
N2—H1*N*···O7^ii^	0.92	2.10	2.817 (3)	134
N2—H2*N*···O3^i^	0.92	2.38	3.028 (3)	127
N2—H2*N*···O4^i^	0.92	2.03	2.938 (3)	169
N4—H3*N*···O2	0.92	2.33	2.734 (3)	106
N4—H3*N*···O4^iii^	0.92	2.12	2.849 (3)	136
N4—H4*N*···O7	0.92	1.84	2.756 (3)	171
C13—H13···F5^i^	1.00	2.38	3.192 (4)	137

Symmetry codes: (i) *x*+1, *y*, *z*; (ii) *x*, *y*, *z*+1; (iii) *x*+1, *y*, *z*−1.
